# Efficacy of live attenuated porcine reproductive and respiratory syndrome virus 2 strains to protect pigs from challenge with a heterologous Vietnamese PRRSV 2 field strain

**DOI:** 10.1186/s12917-018-1451-y

**Published:** 2018-04-19

**Authors:** Tatjana Sattler, Jutta Pikalo, Eveline Wodak, Sandra Revilla-Fernández, Adi Steinrigl, Zoltán Bagó, Ferdinand Entenfellner, Jean-Baptiste Claude, Floriane Pez, Maela Francillette, Friedrich Schmoll

**Affiliations:** 1 Institute for Veterinary Disease Control, AGES, Robert-Koch-Gasse 17, 2340 Mödling, Austria; 2 Clinic for Ruminants and Swine, University of Leipzig, An den Tierkliniken 11, 04103 Leipzig, Germany; 3 Veterinary Practice Entenfellner, Bonnleiten 8, 3073 Stössing, Austria; 4 BioSellal, Bâtiment Accinov, 317 avenue Jean Jaurès, 69007 Lyon, France

**Keywords:** HP PRRSV 2, Vaccine, Efficacy, Viral replication, Immune response

## Abstract

**Background:**

Effective vaccines against porcine reproductive and respiratory syndrome virus (PRRSV), especially against highly pathogenic (HP) PRRSV are still missing. The objective of this study was to evaluate the protective efficacy of an experimental live attenuated PRRSV 2 vaccine, composed of two strains, against heterologous challenge with a Vietnamese HP PRRSV 2 field strain. For this reason, 20 PRRSV negative piglets were divided into two groups. The pigs of group 1 were vaccinated with the experimental vaccine, group 2 remained unvaccinated. All study piglets received an intranasal challenge of the HP PRRSV 2 on day 0 of the study (42 days after vaccination). Blood samples were taken on days 7 and 21 after vaccination and on several days after challenge. On day 28 after challenge, all piglets were euthanized and pathologically examined.

**Results:**

On days 7 and 21 after vaccination, a PRRSV 2 viraemia was seen in all piglets of group 1 which remained detectable in seven piglets up to 42 days after vaccination. On day 3 after challenge, all piglets from both groups were positive in PRRSV 2 RT-qPCR. From day 7 onwards, viral load and number of PRRSV 2 positive pigs were lower in group 1 than in group 2. All pigs of group 1 seroconverted after PRRSV 2 vaccination. PRRSV antibodies were detected in serum of all study pigs from both groups from day 14 after challenge onwards. In group 2, moderate respiratory symptoms with occasional coughing were seen following the challenge with HP PRRSV 2. Pigs of group 1 remained clinically unaffected. Interstitial pneumonia was found in four piglets of group 1 and in all ten piglets of group 2. Histopathological findings were more severe in group 2.

**Conclusions:**

It was thus concluded that the used PRRSV 2 live experimental vaccine provided protection from clinical disease and marked reduction of histopathological findings and viral load in pigs challenged with a Vietnamese HP PRRSV 2 field strain.

## Background

The porcine reproductive and respiratory syndrome (PRRS), caused by PRRS virus (PRRSV), is of great importance in the pig industry worldwide. Recently [1] the PRRSV has been divided into PRRSV 1, the former genotype 1 (European strain, Lelystad virus) [2] and PRRSV 2, the former genotype 2 (North American strain) [3], both of which are of high genetic variability [4]. Highly pathogenic (HP) PRRSV 2 strains have caused great economic losses in Asia, beginning with an especially extensive outbreak with a high mortality not only in piglets but also in sows 2006 in China [5]. Since then, a lot of different subtypes of HP PRRSV 2 have been described [6–8]. Less virulent (non-HP) PRRSV 2 variants, some of them having already been detected in 1996, were reported to occur in Asian countries as well [9].

Because of the wide distribution and the high morbidity and mortality caused by HP PRRSV 2 strains in Asia, efficient immunization strategies are necessary to minimize problems in affected farms. Modified live vaccines often proved to be effective in controlling the infection with PRRSV 1 or (non-HP) PRRSV 2 by reducing the viral shedding and protection against re-infection [10–12]. In many cases, however, commercial vaccines are not as effective as necessary. This can on one hand be caused by the ability of PRRSV to modulate the immune response [13] and is on the other hand due to the high genetic variability of the virus [14]. Unsatisfactory results were especially seen after infection with heterologous virus, where only partial protection could be achieved [15]. PRRSV 2 vaccination with a homologous vaccine conferred better protection, especially against HP PRRSV 2 [16, 17]. Until now, the most effective protection against infection with HP PRRSV 2 was provided by attenuated HP PRRSV 2 vaccines in experimental challenge studies [18]. It is assumed that the highest benefit from vaccination occurs when the vaccine virus is genetically as close to the field virus as possible, as was reported in a study on a homologous attenuated PRRSV 2 live vaccine in China [19]. Another aspect would be the special induction of cellular immunity which has been tried with a homologous DNA vaccine [20].

There is, however, no commercially available vaccine on the market that is able to protect efficiently against infection with HP PRRSV 2 in Vietnam. For any live vaccine it is necessary to identify a batch which is both safe and highly effective in inducing a protective immune response. The aim of this study was to evaluate the potential suitability efficacy of an experimental vaccine containing two live attenuated PRRSV 2 strains in protecting pigs from challenge with a low-passage Vietnamese HP PRRSV 2 field isolate by studying the clinical symptoms, growth parameters, the viral replication and development of antibodies against PRRSV 2. The response to the challenge was compared to not pre-vaccinated pigs.

## Methods

### Experimental design, animals and housing

Twenty male piglets (landrace and large white crossbreds) from a PRRSV 1 and 2 negative farm were selected during the suckling period, marked with an individual ear tag and randomly divided into two groups of 10 piglets each. All piglets were routinely vaccinated twice against Mycoplasma hyopneumoniae (2 ml i.m., Hyoresp, Merial, Halbergmoos, Germany) at the age of 5 and 21 days and against PCV-2 (1 ml i.m., Ingelvac Circoflex, Boehringer Ingelheim, Germany) at the age of six weeks. At the age of 21 days, ten piglets (group 1) were housed in the experimental stable, sized 12 m^2^. Another ten piglets (group 2) were housed in a separate room of the experimental unit with the same size. The units were cleaned daily by qualified personnel. The piglets had permanent free access to drinking water, playing and nuzzling material and were fed ad libitum by an automatic feeder with commercial nursery piglet diet containing colistin sulfate (10 mg/kg body weight, Colistin Mix, AniMed Service AG, Dobl, Austria), amoxicillintrihydrate (20 mg/kg body weight, Amoxi-Mix 10%, AniMed Service AG) and 100 mg zinc oxide/kg body weight (Vetzink®, approved special import from Denmark by Chevita, Wels, Austria) per day. After an adaptation period of five days (day − 42 of the experiment), all piglets of group 1 received an intramuscular injection of 2 ml of a re-suspended experimental vaccine made of two PRRSV 2 strains, containing 10^5^ 50% tissue culture infective dose (TCID_50_) of each strain per dose (strains kindly provided by Kyoto Biken Laboratories, Inc., Kyoto, Japan). This corresponds to a viral load of 1.38E + 09 copies/ml, as determined by reverse transcription quantitative real-time polymerase chain reaction (RT-qPCR). At approximately ten weeks of age (42 days after vaccination, day 0 of the experiment) all piglets of both groups received an intranasal challenge of 2 ml of the challenge virus, an HP PRRSV 2 field strain as described below.

All piglets underwent a daily clinical examination (through visual examination). Blood samples were taken from the piglets of group 1 on days − 42, − 35, − 7 and from piglets of both groups on days 0, 3, 7, 10, 14 and 28 of the experiment. Rectal body temperature of each piglet was measured on the blood sampling days. On days 0 and 28 the pigs were weighed and the weight gain was calculated. Housing, animal care and experimental protocol of the study were approved by the local ethics committee (Agency of the Government in Lower Austria, Department of Agrarian Law).

### Virus strain, titration, calculation of TCID_50_

The HP PRRSV 2 strain Vietnam_PRRSV_AGES/568-30FC/13 (GenBank accession number KM588915, in the following called “challenge virus”) was isolated from serum of a naturally infected pig from a Vietnamese farm, in which severe clinical symptoms of PRRS and a high mortality among pigs were evident. This strain had been identified as HP PRRSV 2 field strain, based on an Nsp2 specific RT-PCR and sequencing [7]. To produce a sufficient quantity of the test virus, the virus was pooled from three consecutive passages in MARC-145 cells over 4 days.

To calculate the infectious PRRSV 2 titer, the Spearman-Karber method was used. PRRSV 2 titers were expressed as TCID_50_/mL. The infectious titer of the virus stock was calculated to be 10^5^ TCID_50_/mL. The PRRSV 2 RNA concentration in the virus stock was 7.28E + 08 copies/ml, as quantified by RT-qPCR.

### RNA extraction and PRRSV ORF7 RT-qPCR

Nucleic acid extraction from serum and tissue samples (lung tissue, pulmonary lymph nodes and tonsillar scrapings) was conducted using the Nucleospin® Virus Core kit and the Nucleospin 96® RNA kit (Macherey-Nagel, GenXpress, Wiener Neudorf, Austria), respectively, on the automated platform Freedom EVO® 150 (Tecan, Grödig, Austria), following the instructions of the manufacturer.

To detect PRRSV 1 and 2 RNA, the samples were analysed by a commercial ORF7 RT-qPCR assay that allows the simultaneous detection and differentiation of PRRSV 1 and 2 (TaqMan® PRRSV Reagents and Controls, Life Technologies, Brunn am Gebirge, Austria) on the ABI 7500 Fast Real-Time PCR System (Life Technologies). For absolute quantification, a PRRSV 2 RNA dilution series with known copy numbers ranging from 1.0E + 00–1.0E + 07 copies/μl was assayed in parallel.

### PRRSV 2 ORF5 amplification and sequencing

The challenge virus stock, the experimental vaccine as well as representative RT-qPCR positive samples collected during the animal experiment (group 1: two serum samples on day − 21, three serum samples on day 7 and nine tonsillar scraping samples on day 28; group 2: four serum samples on day 7 and three tonsillar scrapings on day 28) were subjected to conventional ORF5 RT-PCR, sequencing and phylogenetic analysis. Due to the genetic diversity of some newly emerged Asian HP PRRSV 2 strains [8], specific primers were applied [6; 7]. The corresponding ORF5 RT-PCR products were separated by gel electrophoresis in 1.5% agarose gels stained with ethidium bromide and DNA bands of the expected sizes were excised from the agarose gel and recovered using the QIAquick® Gel Extraction Kit (Qiagen, Hilden, Germany). Sequencing reactions were performed in both directions using the same primers as for ORF5 RT-PCR and the BigDye® Terminator v3.1 Cycle Sequencing Kit (Life Technologies). Sequencing reactions were purified with the DyEx® 2.0 Spin kit (Qiagen). Purified sequencing reactions were resolved on the 3130xl Genetic Analyzer (Life Technologies) and sequence raw data was created with the Data Collection Software (version 2.0, Applied Biosystems, Life Technologies). The raw sequence data was assembled and the consensus sequences were generated using SeqScape Software (version 2.5, Applied Biosystems, Life Technologies). A multiple sequence alignment was done in BioEdit [21], followed by Neighbour joining tree construction (Maximum Composite substitution model, complete deletion of gaps, 1000 bootstrap iterations) using MEGA5 [22].

### PRRSV 2 next generation sequencing (NGS)

Two serum samples from group 1 pigs taken 21 days after vaccination and four samples (two serum and two tonsillar scrapings) from the same group taken 3, 7 and 28 days, respectively, after challenge were selected for NGS. Additionally, the experimental vaccine and the challenge strain were tested.

Prior to NGS, RNA samples were again tested for PRRSV 1 and 2 using the real-time PCR diagnostic assay Bio-T kit® PRRSV (Biosellal, Lyon, France). Total RNA was converted to cDNA and amplified with a combination of one-step and two-step reverse transcription polymerase chain reaction (RT-PCR). One-step RT-PCRs were performed with the One Step RT-PCR kit (Qiagen). Two-step RT-PCRs were performed with the SuperScript® III First strand kit (Invitrogen, Carlsbad, USA) and the Kapa LongRange HotStart PCR kit (Kapa, Wilmington, USA).

Libraries were prepared using the Ion Xpress™ Plus Fragment Library Kit for AB Library Builder™ System (Life Technologies) according to manufacturer’s instructions. The obtained libraries were sequenced by the Ion Torrent PGM sequencer using the 316v2 chip (Life Technologies). Fastq files were analyzed with CLC Genomics Workbench 7.5.1 software (Qiagen). Briefly, reads were trimmed (default parameters) then mapped to the PRRSV strain VR2332 sequence (GenBank No. EF536003.1) with the NGS Reference Assembly tool (default parameters). Alignments and phylogenetic analysis (Neighbour joining, Kimura80, 1000 bootstraps) were all performed with CLC.

### PRRSV antibody ELISA

The presence of PRRSV antibodies in serum from all piglets on each sampling day was assessed by ELISA (IDEXX PRRS X3, IDEXX, Westbrook, USA) following the instructions of the manufacturer.

### Necropsy and histopathology

On day 28 of the experiment, all piglets were narcotized by intramuscular application of Azaperone (2 mg/kg body weight) and Ketamine (20 mg/kg body weight) and then euthanized by intracardial application of 5 ml T61®. Necropsy was performed on all 20 pigs with the main focus on pulmonary lesions and pulmonary lymph nodes. Gross pulmonary lesions were semi-quantified using a scoring scheme after Halbur et al. [23].

For histologic investigation, tissue samples from lungs (cranial and caudal lobe) and pulmonary lymph nodes were taken and fixed in 7,5% neutral buffered formalin. After embedding in paraffin, 4 μm sections were cut and routinely stained with hematoxylin and eosin (HE) and evaluated by light microscopy. Histopathological lung alterations were clustered/quantified according to the scoring scheme as previously described [23] using the following criteria: 0 = no histological alterations, 1 = mild interstitial pneumonia, 2 = moderate multifocal interstitial pneumonia, 3 = moderate diffuse interstitial pneumonia and 4 = severe interstitial pneumonia. Tonsillar scrapings and tissue samples of lung and pulmonary lymph nodes from each piglet were prepared for detection of PRRSV 2 RNA.

### Statistical analysis

Data were tested for normal distribution with the Kolmogorov-Smirnov-test. Since most parameters were not normally distributed, differences between the groups were tested with the Mann-Whitney-U-test. Differences between the sampling times were assessed with the Friedman’s variance analysis test followed by the Wilcoxon test. In cases with more than two sampling times (as was the case in viral load tested by PRRSV 2 RT-qPCR and body temperature) a correction of the alpha error of the significance value was done. Differences of the outcomes of PRRSV 2 RT-qPCR and ELISA and the occurrence of histologic lesions between the groups on each time point were tested with the Fisher’s exact test. Differences with a *P* < 0.05 were considered significant. Correlations between parameters were tested with the rank correlation after Spearman. The correlation coefficient r was indicated in the text if a correlation was found.

## Results

### Clinical data

At the beginning of the experiment, all piglets appeared clinically healthy. After PRRSV 2 vaccination, a slight decrease of appetite was observed for a few days in most of the piglets of group 1. After challenge, pigs of group 1 remained clinically unaffected. Piglets of group 2 showed decreased appetite for a few days after challenge. In most piglets of group 2, occasional coughing and slightly increased lacrimation were observed from day 14 onwards. In two cases, cyanoses on ears, tail and scrotum were seen. These symptoms disappeared after two days. The rectal body temperature did neither increase after vaccination nor after challenge and did not differ between the groups. No significant differences in body weight and weight gain from day 0 to day 28 were detected between the groups.

### PRRSV 2 RT-qPCR and ORF5 sequencing

**Fig. 1:**
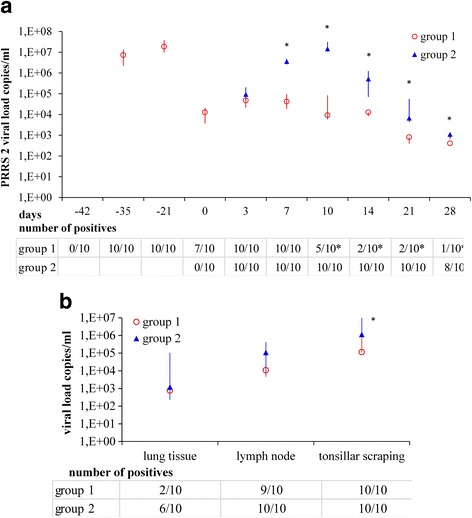
PRRSV loads (copies/ml) in serum (**a**) and tissue samples (**b**) of the study piglets. Blood sampling points were: before vaccination (day − 42), days − 35, − 21, 0, 3, 7, 10, 14, 21 and 28 after challenge with an HP PRRSV 2 field strain; tissue sample were collected on day 28. Data are given as median, 1st and 3rd quartile. Group 1: pre-vaccinated with a new PRRSV 2 live vaccine, group 2: not pre-vaccinated. On time points marked with *, differences between the groups were significant

All study piglets tested negative by PRRSV 1 and 2 RT-qPCR at the beginning of the experiment. The piglets of group 1 tested positive on day − 35 (day 7 after vaccination), which was the first sampling day after vaccination. On day 3 after challenge with HP PRRSV 2, all piglets of both groups tested positive in PRRSV 2 RT-qPCR. Viral loads and number of positive piglets per group on the respective days is shown in Fig. 1a. From day 7 onwards, viral load in the serum of the positive piglets was significantly higher in group 2 than in group 1. The number of PRRSV2 positive pigs was significantly higher in group 2 than in group 1 from day 10 onwards. The PRRSV 2 load in the tissue samples and the number of positive samples are shown in Fig. 1b. In both groups, median viral loads were highest in tonsillar scrapings, followed by lung lymph nodes and lung tissue. In group 1, fewer piglets tested positive in lung tissue than in group 2, although this difference was not statistically significant. Although all piglets tested positive by PRRSV 2 RT-qPCR in tonsillar scrapings on day 28, the viral load in tonsillar scrapings was significantly lower in group 1 than in group 2. There was a positive correlation between PRRSV 2 loads in serum on day 28 and lung and lung lymph nodes. Viral load in tonsillar scrapings was positively correlated with that in serum on days 7, 10, 14, 21 and 28.

**Fig. 2:**
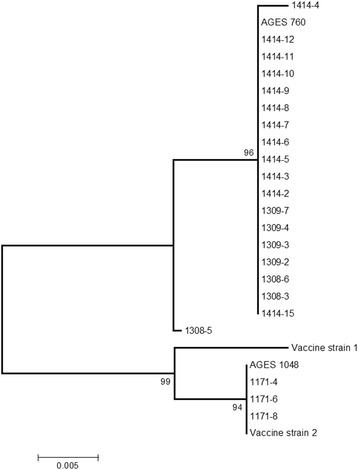
Neighbour joining tree based on partial ORF5 sequences. Obtained from samples of the study piglets, the challenge virus (AGES 760) and the tested experimental PRRSV 2 live vaccine (AGES 1048). Sequences from the two virus strains included in the experimental vaccine (Vaccine strain 1 and 2) were kindly provided by Kyoto Biken. The size of the alignment was 218 bp. Numbers along the branches show the percentage of 1000 bootstrap iterations

Both strains of the experimental vaccine (sequences kindly provided by Kyoto Biken) group within the same cluster as AGES 1048, which was amplified directly from the vaccine (Fig. 2). Sequencing of the ORF5 amplified from four serum samples of group 1 collected on day − 21 showed 100% nucleotide sequence identity to the experimental vaccine strain AGES 1048. On days 7 and 28 after challenge, ORF5 sequences in all sequenced samples from both groups were identical or almost identical to the challenge virus sequence (fig. 2). Sample 1308–5, taken 7 days after challenge, was most distant to the challenge virus (a difference of four nucleotides, equal to 98% sequence identity). In contrast, experimental vaccine and challenge virus only showed 91% sequence identity in the ORF5 region used for comparison (218 bp).

### PRRSV NGS

**Fig. 3:**
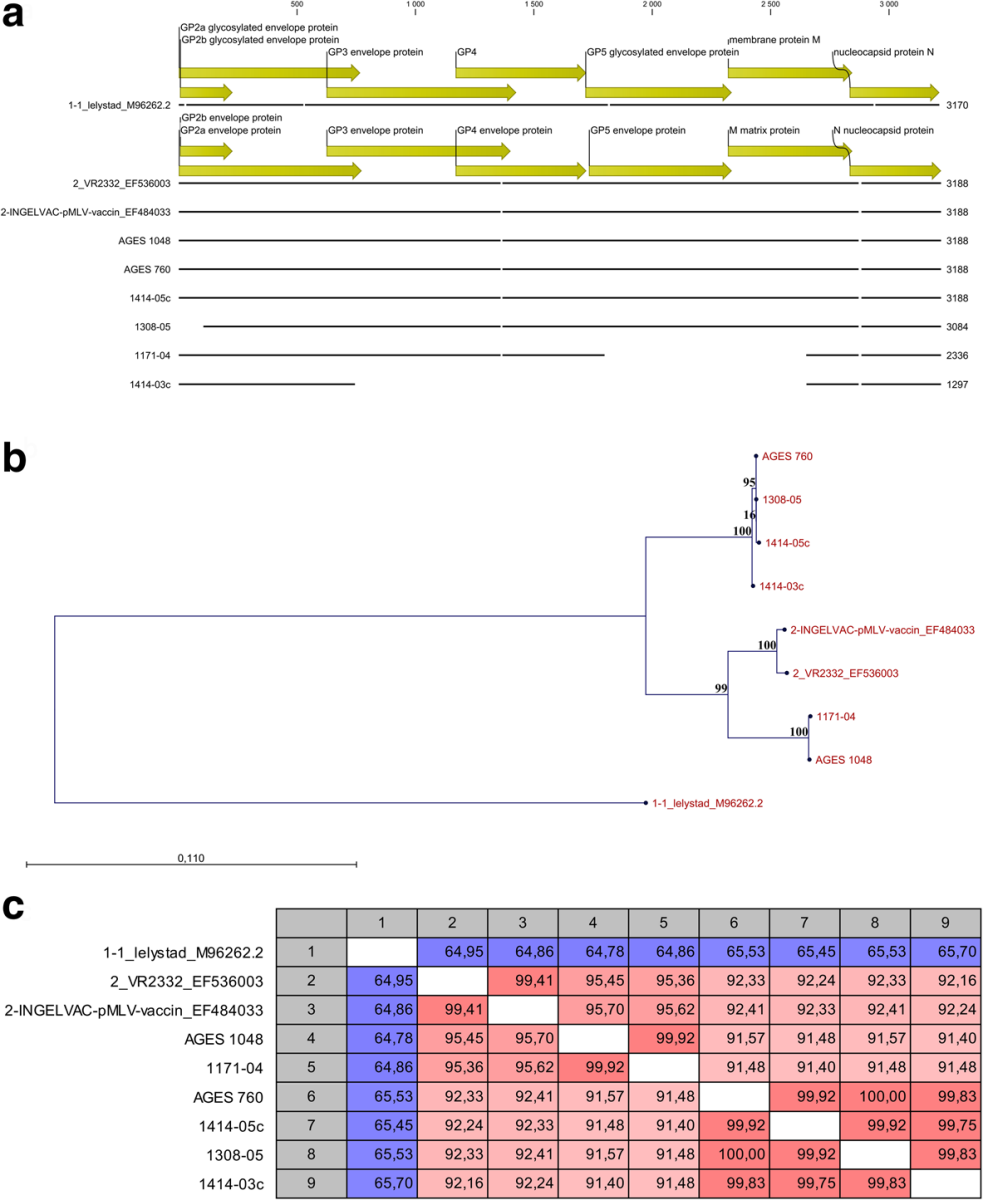
Multiple alignment (**a**), neighbour joining tree (**b**) and nucleotide sequence identity matrix (**c**). Graphical view for selected references, sequenced samples of the study piglets, the challenge virus and the tested PRRSV 2 live vaccine. Reference strains: Lelystad virus for PRRSV 1, INGELVAC pMLV and VR2332 for PRRSV 2. The size of the alignment was 1201 bp. Numbers along the branches in (**b**) show the bootstrap values (%) after 1000 bootstrap iterations

Samples subjected to NGS are listed in Table 1. A graphical view of a multiple alignment between all tested samples and three reference sequences is shown in Fig. 3a. Not all samples could be sequenced over the entire ORF2 to ORF7 region. In sample 1171–04 (21 days after vaccination), the ORFs 5–6 are missing. In sample 1414-3c (tonsillar scraping 28 days after vaccination), ORFs 3–6 are missing. From samples 1171–06 and 1302–01 no sequence could be obtained. Figure 3b and c show the distance tree and the nucleotide sequence identity in the tested samples. Nucleotide sequence identity between the experimental vaccine and the challenge virus was 91.57%. The viral sequences generated from the sample taken before challenge was > 99.9% identical to the experimental vaccine virus, whereas all sequences obtained from post-challenge samples were > 99.8% identical to the challenge virus (Fig. 3). Thus, NGS confirmed the results obtained by partial sequencing of the ORF5 region and corroborated that all viral sequences recovered from post-challenge samples were derived from the challenge virus (Fig. 3).

**Table 1:** Samples selected for next generation sequencing

Name	Sample identity	Sample type	RT-qPCR cq
AGES 1048	PRRSV 2 live vaccine	vaccine	19
AGES 760	HP PRRSV 2 challenge strain	cell culture	20
1171–04	day 21 after vaccination, ear tag 104	serum	29
1171–06	day 21 after vaccination, ear tag 106	serum	26
1302–01	day 3 after challenge, ear tag 101	serum	32
1308–05	day 7 after challenge, ear tag 106	serum	30
1414-3c	day 28 after challenge, ear tag 104	tonsillar scraping	32
1414-5c	day 28 after challenge, ear tag 106	tonsillar scraping	30

All of the selected piglets were pre-vaccinated with an experimental PRRSV 2 live vaccine

### PRRSV antibody ELISA

All piglets were PRRSV antibody negative at the beginning of the experiment. Nine out of the ten piglets of group 1 had seroconverted by day 21 after vaccination. The S/*P* value of the remaining piglet was slightly beneath the test cut-off. On day 0, PRRSV antibodies were present in all piglets of group 1 and in no piglet of group 2. All piglets were PRRSV antibody positive on day 14 after challenge (Table 2).

**Table 2:** Results of PRRSV antibody ELISA in the study piglets

	No. of positive piglets								
Study day	−42	−21	0	3	7	10	14	21	28
Group 1	0	9	10	10	10	10	10	9	9
Group 2	0		0	0	0	6	10	10	10

Group 1 – pre-vaccinated with an experimental PRRSV 2 live vaccine

Group 2 – not pre-vaccinated

### Gross pathology and histopathology

**Fig. 4:**
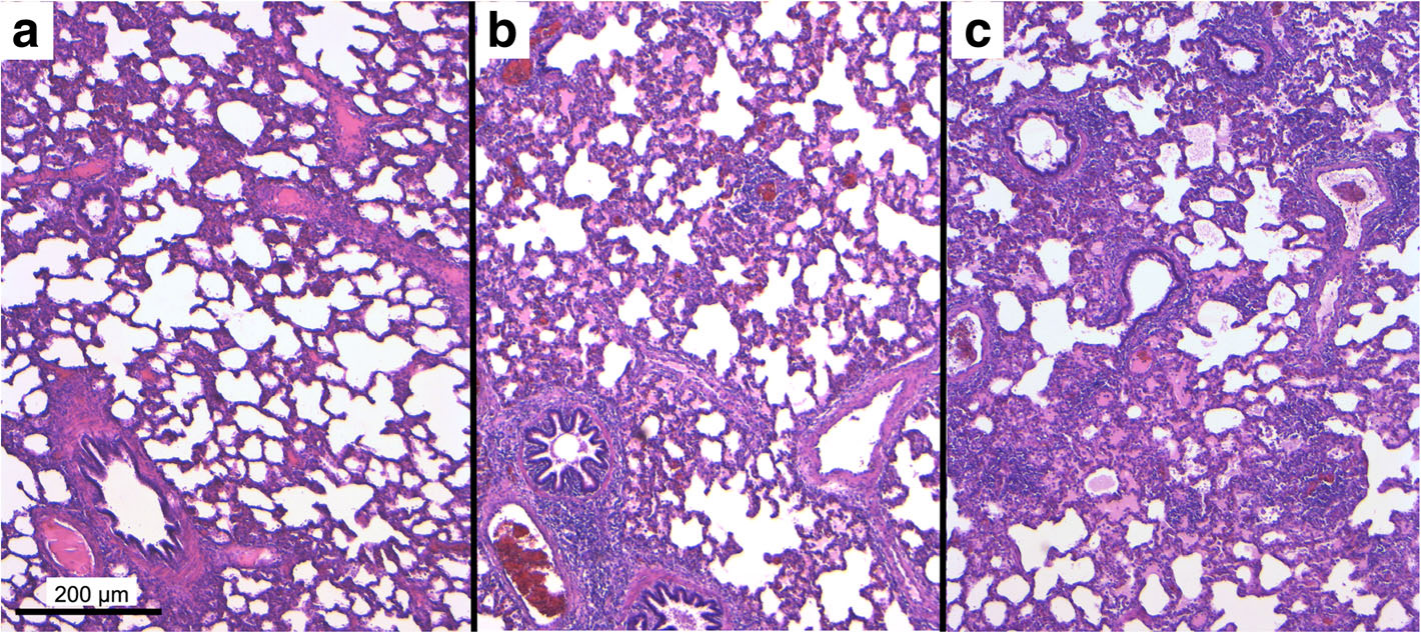
Pulmonary lesions in pigs challenged with an HP PRRSV 2 field strain. **a**: pig from group 1 (vaccinated) without inflammatory alterations (score 0); **b**: pig from group 2 (non-vaccinated) showing moderate multifocal lymphohistiocytic interstitial pneumonia with peribronchial and perivascular accentuation (score 2); **c**: pig from group 2 displaying moderate diffuse lymphohistiocytic interstitial pneumonia with peribronchial, perivascular and intralobular accentuation (score 3). Microphoto; H&E-staining; Bar = 200 μm

An induration of the pulmonary parenchyma was found in eight piglets of group 1 and all piglets of group 2. In all piglets, pulmonary lymph nodes were at least moderately enlarged, however, piglets of group 2 had a more pronounced and generalized lymph node enlargement. Histologically, lymphatic hyperplasia was found in all piglets, which was again more pronounced in the piglets of group 2. An overview of the lung histology results is shown in Table 3. An interstitial pneumonia (intralobular as well as peribronchial) occurred significantly more often in group 2. Gross and histological pulmonary lesions due to lymphohistiocytic interstitial pneumonia were significantly more severe in group 2 as can be seen in Table 4. In Fig. 4, representative microphotos from the lungs of three affected pigs are shown. Overall, the histopathological findings indicated more severe lesions in group 2.

**Table 3:** Histopathological pulmonary findings in pigs challenged with an HP PRRSV 2 field strain (*n* = 10 per group)

	No. of piglets group 1	No. of piglets group 2
Interstitial pneumonia	4^a^	10^b^
Alveolar histiocytosis	4	7
Desquamative/purulent bronchitis	3	7
Dystelectasis	8	10

Group 1 – pre-vaccinated with an experimental PRRSV 2 live vaccine

Group 2 – not pre-vaccinated

Significant differences (*P* < 0.05) between the groups are indicated with different letters

**Table 4:** Score of gross and histological pulmonary lesions due to interstitial pneumonia modified after Halbur et al. [23] in pigs challenged with an HP PRRSV 2 field strain (n = 10 per group) (Median (1st; 3rd quartile))

	Group 1	Group 2
Gross pulmonary lesions	14.5 (9.0; 20.8)^a^	36.5 (26.5; 57.3)^b^
Interstitial pneumonia	1.5 (1.0; 2.0)^a^	3.0 (2.0; 3.0)^b^

Group 1 – pre-vaccinated with an experimental PRRSV 2 live vaccine

Group 2 – not pre-vaccinated

Significant differences (*P* < 0.05) between the groups are indicated with different letters

## Discussion

In this study we assessed the efficacy of two live attenuated PRRSV 2 strains to protect piglets from challenge with a heterologous HP PRRSV 2 field strain (Vietnam_PRRSV_AGES/568-30FC/13; GenBank: KM588915) that had initially been isolated in 2013 from pig serum from a Vietnamese farm.

Although the challenge strain was molecularly typed as highly pathogenic based on a deletion in the Nsp2 region [7], the clinical symptoms upon challenge of unvaccinated piglets (group 2) were moderate. One reason for this could be intrinsic low pathogenicity of the challenge virus. HP PRRSV 2 strains with different pathogenicity in animal experiments have been described [24, 25]. A recent study referred about different pathogenicity of HP PRRSV type 2 strains isolated from Northern and Southern Vietnam, with higher pathogenicity of the Northern strain [26]. However, the challenge virus likely caused severe clinical symptoms and a high mortality in the Vietnamese farm where it was isolated from. The relatively mild symptoms observed in unvaccinated piglets might also be due to the fact that the animals were healthy at the time of challenge and bacterial infections were prevented by the use of antibiotics throughout the study. Furthermore, tests for antibodies against classical swine fever, Aujeszky’s disease, swine influenza virus and *Actinobacillus pleuropneumoniae* as well as *Mycoplasma hyopneumoniae* DNA at day 28 were negative in all study piglets (data not shown). Since the challenge virus remained genetically stable during the three cell culture passages, it is unlikely that the mild clinical symptoms are due to a genetic attenuation of the virus. Attenuation can only be expected after several passages [27]. In other studies low passages were successfully used for challenge as well [28].

In piglets of group 1 (vaccinated), no clinical symptoms were seen after PRRSV challenge. This is in line with the significantly milder gross pulmonary lesions and histopathological findings compared to group 2 and proves the efficacy of the tested vaccine to prevent clinical symptoms and diminish pathological lesions after infection with the heterologous HP PRRSV 2 challenge virus. As determined by NGS, experimental vaccine viral strains and challenge virus only shared 91.57% nucleotide sequence identity over the entire ORF2 – ORF7 region. Studies testing the efficacy of the vaccine strains used in this study after challenge with HP PRRSV 2 are not available. In a study of Wei et al. [16], clinical symptoms after HP PRRSV 2 challenge could not be prevented but diminished using a PRRSV 2 attenuated live vaccine. The control group in their study, however, developed severe clinical symptoms after challenge. Similar results were found in other studies [17, 29, 30].

Intranasal challenge with the defined PRRSV dose resulted in detectable virus replication in all study piglets on the first day of sampling after challenge (day 3). In the unvaccinated group 2, high viral loads were detected in serum, lung, pulmonary lymph nodes and tonsillar scrapings. This proves a rapid virus replication in the unvaccinated piglets. The viral loads were comparable to those found by Hu et al. [31] and Han et al. [32] after challenge with HP PRRSV type 2 strains isolated in China. In the vaccinated pigs (group 1), the viral load in serum as well as the number of viraemic piglets were significantly lower than in group 2. Lager et al. [17] describe similar levels of protection conferred by a homologous HP PRRSV 2 vaccine, using virus isolation as readout instead of PCR. This further underlines the efficacy of the experimental vaccine tested in our study. In a study using different dosages of an HP PRRSV 2 vaccine, the experimental vaccine was able to protect the study pigs from viraemia after homologous challenge when administered at least at the two-fold dosage used in our study [28]. Other studies, using heterologous PRRSV vaccines, also referred about partial protection (fewer clinical symptoms and viraemia compared to non-vaccinated pigs) against challenge with HP PRRSV [29, 30].

NGS was chosen to sequence a larger part of the experimental samples in a cost-effective way and to verify the results from Sanger sequencing, especially because one post-challenge sequence from group1 (sample No 1308–05) differed from the remaining post-challenge sequences. To investigate whether this sample might be a recombinant between vaccine and field virus, NGS was applied to obtain (a) a longer stretch of sequence to improve identification of potential recombination breakpoints; (b) to obtain a higher coverage of the sequence in question. In some cases, not the entire ORF2 – ORF7 sequence could be obtained by NGS, or sequencing failed completely. This is probably due to the relatively high Cq value in some of these samples. In cases of samples with cq values above 25, the success of NGS diminishes as has been described in another study [33].

The HP PRRSV 2 challenge strain used in this study remained genetically stable not only during replication in cell culture but also during the animal experiment as shown by both partial sequencing of the ORF5 and NGS of the ORFs 2 to 7. The experimental vaccine strain found in the pigs remained genetically stable as well during the animal experiment. Furthermore, there was no evidence of recombination between experimental vaccine and challenge virus, as all viral sequences obtained from experimental animals before and after challenge were more than 99% identical to the experimental vaccine and challenge virus sequence, respectively.

The humoral immune response to the tested vaccine is shown by detection of PRRSV antibodies by ELISA in all vaccinated piglets (group 1) on day 21 after vaccination. Lager et al. [17] obtained similar results, whereas others [16, 30] report that on day 21 after PRRSV 2 live vaccination, only a part of the pigs was PRRSV antibody positive by ELISA. In group 2, all piglets had developed PRRSV antibodies on day 14 after challenge. In other studies, PRRSV antibodies were found on this time point after challenge as well [34, 35].

## Conclusions

Vaccination with new live attenuated PRRSV 2 strains induced an immune response as shown by timely production of PRRSV antibodies. Experimental infection with the heterologous HP PRRSV 2 challenge virus resulted in viraemia in all study piglets that was significantly lower in animals vaccinated with the experimental vaccine. Although PRRSV loads in serum and tissues of the unvaccinated study piglets were high, the development of clinical symptoms was moderate. Nevertheless, histological findings indicated interstitial pneumonia and/or other pulmonary lesions in all of the unvaccinated piglets. No clinical symptoms and less severe pathological findings were seen in the vaccinated piglets. Thus, the tested live attenuated PRRSV 2 strains were able to provide an efficient partial protection against heterologous challenge with a Vietnamese HP PRRSV 2 field strain.

## Abbreviations

HP: Highly pathogenic
NGS: Next generation sequencing
PRRSV: Porcine Reproductive and respiratory disease virus
RT-qPCR: Reverse transcription quantitative real-time polymerase chain reaction
TCID_50_: 50% tissue culture infective dose
